# The PinkThing for analysing ChIP profiling data in their genomic context

**DOI:** 10.1186/1756-0500-6-133

**Published:** 2013-04-04

**Authors:** Fiona G Nielsen, Maarten Kooyman, Philip Kensche, Hendrik Marks, Henk Stunnenberg, Martijn Huynen

**Affiliations:** 1 CMBI - Centre for Molecular and Biomolecular Informatics, Nijmegen Centre for Molecular Life Sciences, PO box 9101, 6500HB Nijmegen, Netherlands; 2 Department of Molecular Biology, Radboud University Nijmegen, Nijmegen Centre for Molecular Life Sciences, PO box 9101, 6500HB Nijmegen, Netherlands

## Abstract

**Background:**

Current epigenetic research makes frequent use of whole-genome ChIP profiling for determining the *in vivo* binding of proteins, e.g. transcription factors and histones, to DNA. Two important and recurrent questions for these large scale analyses are: 1) What is the genomic distribution of a set of binding sites? and 2) Does this genomic distribution differ significantly from another set of sites?

**Findings:**

We exemplify the functionality of the PinkThing by analysing a ChIP profiling dataset of cohesin binding sites. We show the subset of cohesin sites with no CTCF binding have a characteristic genomic distribution different from the set of all cohesin sites.

**Conclusions:**

The PinkThing is a web application for fast and easy analysis of the context of genomic loci, such as peaks from ChIP profiling experiments. The output of the PinkThing analysis includes: categorisation of position relative to genes (intronic, exonic, 5’ near, 3’ near 5’ far, 3’ far and distant), distance to the closest annotated 3’ and 5’ end of genes, direction of transcription of the nearest gene, and the option to include other genomic elements like ESTs and CpG islands. The PinkThing enables easy statistical comparison between experiments, i.e. experimental versus background sets, reporting over- and underrepresentation as well as p-values for all comparisons. Access and use of the PinkThing is free and open (without registration) to all users via the website: http://pinkthing.cmbi.ru.nl

## Findings

### Background

Transcription factor (TF) binding sites can be identified *in vivo* using the emerging technologies for ChIP profiling such as ChIP-on-chip and ChIP-sequencing. These experiments locate hundreds to tens of thousands TF binding sites, which subsequently have to be validated and analysed for biological function. Certain initial analyses of TF binding sites have already become commonplace: mapping of the binding sites to the genome, detecting closest genes to the binding sites, categorising binding sites by their position relative to the genes, and the subsequent GO analysis of the genes closest to the binding sites. The same type of analyses apply to other regions found by ChIP profiling, e.g. with specific histone modification patterns or DNA hyper- or hypomethylated loci. The PinkThing gathers all these analyses into one single user-friendly tool, standardises the characterisation of genomic locations, uses up-to-date ENSEMBL gene annotation, and at the same time enables statistically sound comparisons at each step of the analysis. Although it is possible to perform similar analysis using bioinformatic packages like Taverna [1] or Galaxy [2], these packages require installation, initialisation and scripting (Taverna) or a certain level of statistics skills from the user (e.g. Galaxy). Another more specialised tool is the HyperBrowser [3], which is based on Galaxy and designed to incorporate functions to query and correlate annotation along the genome. The versatility of this tool is impressive, but it comes at the cost of usability. Compared to the PinkThing, the HyperBrowser has a complex interface that takes time to learn to use efficiently. A more accessible tool is GREAT [4] which provides a web interface for analysis, with a focus specifically on cis-regulatory regions for human, mouse and zebrafish.

With the PinkThing tool the genomic analyses with statistics are available and ready-to-use directly from the website by a simple upload of a file containing the coordinates of genomic regions of interest. In addition, the PinkThing provides the option of supplying an appropriate background distribution to be used for comparisons, e.g. the set of all binding sites of a ChIP-seq experiment when examining a subset of these sites. Furthermore, the PinkThing analysis of genomic distributions supports all species that are annotated in Ensembl. PinkThing has already been successfully used for the analysis of ChIP profiling data in a wide variety of data, including [5-17].

### Approach

**Figure 1:**
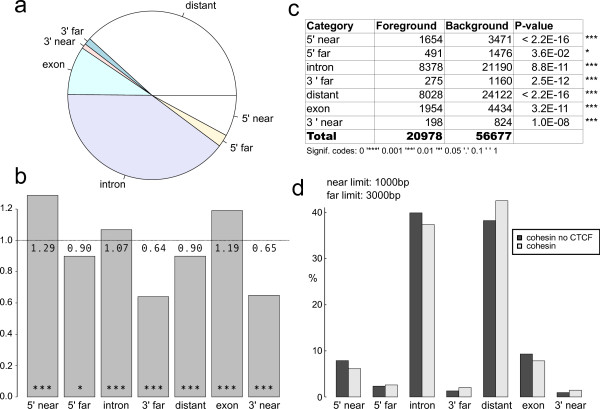
**PinkThing produces graphical output along with the statistics of the genomic distributions.** Three plots and a table produced by the PinkThing comparison of Cohesin sites (background) versus Cohesin-no-CTCF sites (foreground). **a**) PinkThing pie chart showing the overall genomic distribution of cohesin-no-CTCF sites directly upon upload. **b**) The output of the PinkThing comparison illustrating the category sizes by ratio of foreground over background, indicating significance by stars as indicated in Figure 1c. **c**) Table of statistics for Cohesin-no-CTCF (foreground) versus all Cohesin sites (background) with the G-test p-value for each genomic category. **d**) The barplot of category frequencies of the compared distributions, showing the relative frequencies side-by-side. For this analysis the ‘near’-limit was set to 1000bp and the ‘far’ limit to 3000 bp.

Through the PinkThing web page, the user uploads the genomic sites of interest using a standard format for genomic locations (BED format). PinkThing compares the sites to the Ensembl gene annotation and optionally to Ensembl CpG islands, ESTs and regulatory features. The initial results include basic statistics of the genomic distribution of the uploaded positions: 

•histograms of distances to the 5’ and the 3’ ends of genes,

•a barplot and a pie chart of the genomic distribution relative to Ensembl genes/CpG islands/ESTs (Figure 1a).

•a barplot of the genomic distribution.

Every output plot is clickable for download of an SVG (Scalable Vector Graphics) version of the plot for publication. Subsequent analyses can be accessed from the results page and include: 

•transcription start sites (TSS) within the surrounding regions,

•the conservation score (GERP [18]) for these regions,

•comparisons to other sets of genomic regions,

•retrieval of the reference DNA sequence for the uploaded regions,

•GO annotation and GO enrichment analysis of the nearest genes (only available for human and mouse).

As an example, we considered the CTCF (CCCTC binding factor) and cohesin binding sites as determined using ChIP-seq in a study by Schmidt *et al.*[19]. In this study, Schmidt *et al.* also discovered a subset of cohesin binding sites that did not overlap with CTCF binding sites. Using the PinkThing we compared the genomic distribution of the set of cohesin sites with the set of cohesin sites not bound by CTCF (cohesin-no-CTCF sites). We chose the cutoffs for ‘near’ and ‘far’ categories in our analysis to be 1000bp and 3000bp respectively, to match the categories presented in their paper [19] and examined PinkThing-determined categorisation and genomic distribution of the sites (Figure 1a). The categorisation of the subset cohesin-no-CTCF is very informative when viewed in relation to the background set of all cohesin binding sites: The PinkThing provides the relative sizes of each of the categories for the two sets of sites (Figure 1b), the Brandt-Snedecor test statistic for comparison of the two distributions, as well as p-values for whether the differences in distributions are significant per category (Figure 1c) as well as a barplot showing the frequencies of the two distributions grouped per category (Figure 1d).

The Brandt-Snedecor test indicates that the genomic distribution of cohesin and cohesin-no-CTCF sites are significantly different (*p*<2.2*e*−16) and the individual category comparison shows the cohesin-no-CTCF sites are significantly overrepresented in promoter regions (5’ near) and exon regions, and underrepresented in 3’ regions as compared to the set of all cohesin sites (Figure 1c). The Gene Ontology (GO) result from the PinkThing (Additional file 1: noCTCF-vs-cohesin.xls) indicates that the cohesin-no-CTCF binding sites are, relative to all cohesin sites, overrepresented (adjusted *p*<10*e*−3) in the following GO categories: nucleic acid binding (GO:0003676), transcription regulator activity (GO:0030528), regulation of metabolic process (GO:0019222) (See Supplementary material: noCTCF-vs-cohesin-GO.xls). The difference in genomic distributions suggests a difference in function, which was confirmed by Schmidt *et al*. who showed that the cohesin-no-CTCF sites are enriched for DNA binding sites of tissue-specific transcription factors, and further explored this fact in a follow-up study [20].

### Methods

#### Database access

Lookup and data retrieval from the Ensembl database was implemented using the Ensembl Perl API http://www.ensembl.org/info/docs/api/core/core\_tutorial.html.

#### Statistics

Statistical tests and generation of plots and figures on the PinkThing website are implemented in R [21] and bioperl [22]. Detailed description of plots and their interpretation is in the PinkThing manual available from the PinkThing website.

#### Categorising positions

For each imported locus, PinkThing determines the distance to the closest gene. Positions that fall into multiple categories are assigned to the highest ranking category in the order: exon, intron, 5’ near, 3’ near, 5’ far, 3’ far and distant. As the surrounding sequence may contain overlapping gene annotations, the gene annotations are merged such that each position corresponds to a unique genome category. The limits for “near” and “far” may be chosen by the user, with default values being 5kb and 25kb, respectively.

#### Comparison of category distributions

The initial statistical test is the Brandt-Snedecor test for homogeneity of k binomial distributions [23]. The test assumes *i)* that the observations are independent; *ii)* that the set chosen as background distribution is at least twice the size as the set chosen as sample distribution and *iii)* that the *k* categories are exhaustive and mutually exclusive. Our implementation of the Brandt-Snedecor test does not test whether there is an overlap of actual observations in the two sets of the comparison. The test compares the distribution of observations over categories, indifferent to the identity of the observation. In our example above we show how we apply the comparison between a set of sites (cohesin binding sites) and a subset of those sites (cohesin-noCTCF sites), but the test can also be applied to compare two disparate sets of sites in the genome.

Let *n*_1_ and *n*_2_ be the total counts for each of the distributions 1 and 2, with *n*=*n*_1_+*n*_2_ and let *n*_2*i*_ and *n*_1*i*_ be the counts within category *i*, then the test statistic is given by Equation 1, and the p-value obtained by comparing to a $\chi_{k-1}^{2}$ distribution. If the distributions are identical, the value of the test will be 1.

(1) $$[H]{\hat{\chi }}^{2}=\frac{n^{2}}{n_{1}\times n_{2}}\left(\left(\overset{k}{\underset{i=1}{\sum }}\frac{{n_{1i}}^{2}}{n_{2i}+n_{1i}}\right)-\frac{n_{1}^{2}}{n}\right)$$

To determine whether each of the genomic categories is significantly overrepresented, we apply the log likelihood G-test for independence [24] per category, comparing each category against the union of the other categories.

#### GO analysis

The PinkThing uses Ontologizer [25] to perform GO statistics on the set of genes that are closest to the uploaded set of sites. The output includes both the graphical Ontologizer visualization of overrepresented categories as well as a table with p-values of all terms. The GO statistics can either be calculated against the whole genome as background or against the genes corresponding to another uploaded set. Currently the GO ontology analysis is available for human and mouse.

## Conclusion

Determination of the annotation context of genomic loci is an indispensable foundation for their functional analysis. To allow distributions of genomic categories to be compared within the same genome assembly, the implementation of categorisation in PinkThing consistently assigns exactly one category to any genomic position, thus providing consistent and reproducible analysis. The PinkThing categorisation into consistent genomic categories allows the comparison with a background or reference distribution. This comparison is essential when examining distributions where the categories vary in size and abundance dependent on the context of the genome (the species) and the experiment (e.g. selecting for specific genomic elements). PinkThing makes it easy to compare results from a specific experiment with a chosen background distribution, thus increasing the value and confidence in interpreting results.

PinkThing is a collection of the most common genomic analyses related to genomic context, combined in a simple point and click web interface. With no prerequisites other than obtaining a dataset, PinkThing provides easy access to sound statistical analysis of genomic location data.

## Availability and requirements

Access and use of the PinkThing is free and open (without registration) to all users via the website: http://pinkthing.cmbi.ru.nl

•**Project name:** The PinkThing for analysing ChIP profiling data in their genomic context

•**Project home page:**http://www.bioinformatics.org/websvn/listing.php?repname=pinkthing

•**Operating systems:** Usage of web tool is platform independent, access is available via all major web browsers at http://pinkthing.cmbi.ru.nl.

•**Code repository (SVN):**http://www.bioinformatics.org/websvn/listing.php?repname=pinkthing

•**License:** GNU GPL

The two data sets with cohesin binding sites with and without CTCF are available for download from the front page of the PinkThing and included with the additional files for this article (Additional file 2: all cohesin binding sites, cohesin.bed; Additional file 3: cohesin binding sites without CTCF, no_CTCF_cohesin.bed).

To reproduce the results in this paper: 

1. Go to http://pinkthing.cmbi.ru.nland select Ensembl version 53.

2. Then enter the desired cutoff for near and far positions as 1000 and 3000 respectively.

3. Upload the two files one at a time by selecting the file location and click ‘upload and calculate’.

4. To perform the comparison, from the start page of the PinkThing, select the two uploaded files in the box ‘Compare genomic distributions’, choosing the cohesin sites with no CTCF binding as the sample file (foreground) and choosing the set of all cohesin binding sites as sample space(background).

5. Click ‘Compare’ to view the results.

6. To find overrepresented ontologies, from the start page of the PinkThing, select the two uploaded files in the respected boxes for Sample space (all cohesin binding sites) and sample file (cohesin binding sites without CTCF) and click Ontologize.

## Competing interests

The authors declare that they have no competing interests.

## Authors’ contributions

FGN and MK developed the PinkThing functionality and web interface. FGN prepared the manuscript. PK reviewed the implementation of statistical functions. HM tested and suggested functionality during preparation of the manuscript. HS and MH supervised the study. All authors read and approved the final manuscript.

## Supplementary Material

Additional file 1 **noCTCF-vs-cohesin-GO.xls.** The Ontologizer result of comparing the cohesin sites without CTCF (the study set) against the background set of all cohesin sites. Click here for file

Additional file 2 **cohesin.bed.** Cohesin binding sites in BED format, as obtained from Schmidt *et al.*[19]. Click here for file

Additional file 3 **No CTCF cohesin.bed.** Sites of cohesin binding with no CTCF binding, as obtained from Schmidt *et al.*[19]. Click here for file
